# No gender differences in the 24-month course of non-invasive liver fibrosis markers after DAA therapy in HCV-mono and HCV/HIV-coinfected patients

**DOI:** 10.1038/s41598-024-57845-x

**Published:** 2024-03-29

**Authors:** Julio Collazos, Laura Pérez-Is, Belén de la Fuente, Luis Morano, Maria Rivas-Carmenado, Manuel Rodriguez, Adolfo Romero-Favela, Galilea de Jesús Fonseca-González, Santiago Melón, Javier Diaz-Arias, Eulalia Valle-Garay, Víctor Asensi

**Affiliations:** 1 Infectious Diseases Unit, Hospital of Galdakao-Usansolo, Galdácano Vizcaya, Spain; 2 Biochemistry and Molecular Biology, University of Oviedo Medical School, Oviedo, Spain; 3 Infectious Diseases Unit, Hospital de Cabueñes, Gijón, Spain; 4 Infectious Diseases Unit, Complejo Hospitalario Universitario de Vigo (CHUVI), Vigo, Spain; 5 Infectious Diseases-HIV Unit, Hospital Universitario Central de Asturias, University of Oviedo Medical School, Oviedo, Spain; 6 Gastroenterology and Hepatology Service, Hospital Universitario Central de Asturias, University of Oviedo Medical School, Oviedo, Spain; 7 Virology Division, Hospital Universitario Central de Asturias, University of Oviedo Medical School, Oviedo, Spain; 8 Universidad Tecnológica de Sinaloa, Sinaloa, México; 9 Group of Translational Research in Infectious Diseases, Instituto de Investigación Sanitaria del Principado de Asturias (ISPA), Oviedo, Spain; 10 Infectious Diseases-HIV Unit, Hospital Universitario Central de Asturias, Avenida de Roma S/N, 33011 Oviedo, Spain

**Keywords:** Gender, Hepatitis C, HIV, Liver fibrosis, Transient elastometry, Direct-acting antivirals, Biomarkers, Diseases, Gastroenterology, Medical research, Pathogenesis, Signs and symptoms

## Abstract

Untreated HCV mono and HCV/HIV coinfected women have lower degrees of liver fibrosis (LF) compared to men. Direct acting antiviral (DAA) therapy attains viral eradication in > 90% of patients with progressive LF decline in parallel. Gender-related differences in LF regression in the long term assessed by non-invasive liver fibrosis markers (NILFM) in HCV mono and HCV/HIV coinfected after DAA treatment have not been explored so far. 374 HCV-infected adult patients, 214 of them HCV/HIV coinfected, were followed-up for 24 months after starting DAA therapy. LF was assessed by NILFM: transient elastometry (TE) and several biochemical indexes (APRI, Forns, FIB-4). Men had significantly more advanced LF at baseline than women assessed by NILFM. No LF differences at baseline in age, HIV coinfection course (CD4, HIV viral load), and HCV features (HCV viral load, genotype) were detected. No significant gender differences in LF decline after comparing 24-month and baseline LF values were observed. LF changes after DAA therapy were similar in HCV mono and HCV/HIV coinfected patients and in both sexes. Gender did not influence the course of LF decline after DAA assessed by NILFM: TE (P = 0.8), APRI (P = 0.9), Forns (P = 0.4) and FIB-4 (P = 0.7) by multivariate analysis. No gender differences in the 24 month LF decline after DAA with independence of having HCV mono or HCV/HIV coinfection were found.

## Introduction

Hepatitis C virus (HCV) infection is a major cause of progressive liver fibrosis (LF) with cirrhosis, liver transplantation and hepatocarcinoma as its late-stage most severe complications^1^. Gender differences in LF in HCV-infected patients have been rarely explored in spite of the nowadays availability of non-invasive liver fibrosis markers (NILFM) including transient elastometry (TE), and biochemical indexes (APRI, Forns, FIB-4) instead of liver biopsy to assess LF^2–5^. Hormonal changes are involved in LF progression in HCV-infected women. Menopause is associated with accelerated LF while pregnancies and estrogen administration are LF-protective^6–8^. A protective effect of female gender on LF, lost after menopausal was reported in Chinese women with chronic hepatitis B infection^9^ On the other hand higher degrees of LF in men are associated with lower free testosterone and higher sex-hormone-binding globulin (SHBG)^10^. Cell-intrinsic androgen receptors could drive CD8^+^ T cell dysfunction and secondarily modulate LF in men as they do in cancer immunotherapy^11^. Different behaviors of men and women including the abuse of alcohol, tobacco or intravenous drugs, more prevalent among male patients, might enhance their HCV-induced LF. HIV infection could contribute to increasing LF in HCV/HIV-coinfected individuals^12–17^. However HCV/HIV coinfected women have lower degrees of LF compared to men with independence of their alcohol consumption^18^. Direct-acting antiviral agents (DAA) are extremely effective in combination and even in monotherapy to treat HCV infection with > 90% efficacy^19^. The dynamics of LF at long term after DAA are not well understood due to the lack of extended follow-up studies and the different tools used for LF assessment^2,10–24^. It seems that LF decreases quickly after starting DAA therapy and then a slow decline or even a plateau in the LF curve is reached. The quick LF decrease has been attributed to early resolution of liver inflammation, although it does not always occur^21^. In 2022 we reported a continuous improvement of LF measured by NILFM in HCV mono and HCV/HIC coinfected patients after DAA therapy and 24 months of follow-up^25^. However in 2023 Gardner et al. observed a lack of decline beyond one year after HCV cure in LF assessed by an enhanced liver fibrosis (ELF) score, APRI and FIB-4 indexes in HCV/HIV coinfected American women^26^.

To our knowledge, no study to date has focused on the evaluation of fibrosis regression according to gender. We hypothesized that because HCV and HCV/HIV coinfected women had lower LF before DAA therapy compared to men, their LF reversal after DAA treatment might be different. This LF regression differences might be more evident after a long term follow-up. On the other hand, the presence of HCV mono or HCV/HIV coinfection could induce different degrees of LF regression.

The aim of this study was to comparatively evaluate the influence of gender in the long-term course of LF decline, as measured by NILFM, in HCV mono- and HCV/HIV coinfected patients treated with different DAA regimens, and to identify other factors that might modify LF regression after DAA therapy such as HIV coinfection. To this end we have carried out a prospective and comprehensive study on a large sample of HCV mono- and HCV/HIV coinfected male and female Spanish patients who were followed-up at regular intervals for 24 months after DAA treatment.

## Patients and methods

### Patients

Patients with active HCV monoinfection or HCV-HIV coinfection demonstrated by positive serology and viral RNA plasma levels were enrolled in the study when starting DAA therapy. Patients were Caucasians, older than 18 years and were recruited from three third level hospitals of Northwest Spain. A number of demographic, epidemiological, laboratory and clinical data were obtained from the patients and from their electronic medical charts. All HCV-HIV-coinfected patients were receiving ART at the inclusion time. The DAA regimens used included NS5B inhibitors (sofosbuvir, dasabuvir), NS3/4A inhibitors (glecaprevir, paritaprevir, asunaprevir, grazoprevir, simeprevir) and NS5A inhibitors (velpatasvir, ledipasvir, daclatasvir, pibrentasvir, ombistavir, elbasvir) with or without ribavirin. DAA were selected according to the attending clinician criteria. LF was assessed by TE (Fibroscan) and by the noninvasive biochemical biomarkers APRI, Forns and FIB-4 at baseline and at the 1st, 3rd, 6th, 12th and 24th months.

### Exclusion criteria

To avoid LF confounding factors different from HCV and HIV infections, patients with HBV coinfection with/out delta virus coinfection, ethanol consumption ≥ 50 g/day for > 5 years, alcoholic hepatopathy, and other liver diseases were excluded from the study as we previously did^27^. Pregnant women and those individuals in whom there were technical difficulties for obtaining reliable TE readings were also excluded. In addition, patients with ascites or spontaneous bacterial peritonitis were excluded because TE reading could be altered by these factors^5,28,29^.

### Transient elastometry

LF was assessed by TE using Fibroscan (EchoSens,Paris, France) following pre-established methods^5,28,29^. Patients were classified into 4 groups following the TE assessments (F0-F1, F2, F3 and F4), reflecting the progressive degree of LF by similarity with the histological stages of the METAVIR grading system. Thus, < 7.2 kPa measurements were considered minimal or no fibrosis (F0-F1), values in the range of 7.2–9.3 kPa as significant fibrosis (F2), those in the range of 9.4–13.9 kPa as advanced fibrosis (F3), and values > 13.9 kPa as cirrhosis (F4)^25,28^.

### Laboratory methods

HIV and HCV serologies were assessed by enzyme immunoassay (MEIA AxSYM; Abbott Diagnostics,Abbott Park, IL, United States). HIV and HCV RNA by quantitative PCR (Cobas TaqMan; Roche Diagnostics, Branchburg, NJ, United States) and HCV genotypes by a line probe assay (Versant HCV, Siemens). Routine laboratory methods were used to calculate three LF indexes: AST and platelets for APRI index^30^, age, platelet counts, total cholesterol and GGT for Forns index^31^, and age, AST, ALT and platelet counts for FIB-4^32^.

### Statistical analysis

Due to the non-Gaussian distribution of the fibrosis markers studied, they underwent natural logarithmic transformation for analysis, and were back-transformed to the original units for reporting. Categorical variables are reported as percentage, continuous variables as mean and 95% confidence interval (CI), and the differences between sexes were assessed by the chi-square test and the t-test, respectively. Paired t-tests were used to compare the changes in the four NILFM between two time points within the same individual. The overall absolute and relative improvements in fibrosis indexes at the end of the follow-up period were calculated by subtracting and dividing, respectively, the values observed at the 24-month respect to the baseline measurements. A multivariate general linear model was elaborated for each of the four fibrosis markers, in order to compare their courses over time to verify if the behavior of these markers was influenced by gender. Statistical calculations were performed with the SPSS software v.25 (IBM Corp., Armonk, NY, USA). The cut-off for statistical significance was established at P < 0.05 for a two-sided test.

### Ethics approval

This study was performed in line with the principles of the Declaration of Helsinki. Approval was granted by the Ethics Committee of the Hospital Universitario Central de Asturias (HUCA).

### Consent to participate

All patients underwent standard of care, including routine noninvasive procedures. Informed consent was obtained from all individual participants included in the study.

## Results

The study included 374 HCV-infected patients, 264 men and 110 women, who underwent DAA therapy and were followed-up for 24 months afterwards, although a total of 85 patients did not accomplish the last evaluation (25.4% of the women and 21.6% of the men). The mean age was 50.8 years (95% CI 50.0–51.6), 75.6% had genotype 1 infection and 57.2% were coinfected with HIV.

Table 1 shows the baseline characteristics of men and women, as well as the absolute and relative changes of the four NILFM at 24 months in comparison with the baseline values. There were no differences in age, HIV coinfection, HCV features and HCV treatment between the two groups. On the contrary, men had significantly higher levels of certain laboratory parameters, such as hemoglobin, γ-glutamyl transferase, bilirubin, creatinine, as well as lower levels of platelets, and total and HDL cholesterol than women.

**Table 1:** Baseline demographic, HIV, HCV, laboratory and fibrosis parameters in men and women.

	Men (n = 264)	Women (n = 110)	P value
Age (years)	50.46 (49.61–51.32)	51.63 (49.92–53.33)	0.2
HCV-related parameters			
HCV viral load (Log copies/mL)	5.865 (5.708–6.022)	5.969 (5.743–6.195)	0.5
HCV genotype			
1	145 (75.9%)	60 (75.0%)	0.9
2	1 (0.5%)	1 (1.3%)	
3	28 (14.7%)	28 (14.7%)	
4	17 (8.9%)	17 (8.9%)	
HCV treatment			
Time on DAA therapy (weeks)	13.06 (12.56–13.57)	13.16 (12.34–13.99)	0.8
Sofosbuvir			
Yes	229 (86.7%)	96 (87.3%)	0.9
No	35 (13.3%)	14 (12.7%)	
Drug combinations			
Sofosb + Velpatasvir	104 (39.4%)	45 (40.9%)	0.9
Sofosb + Ledipasvir	101 (38.3%)	40 (36.4%)	
Other combinations	59 (22.3%)	25 (22.7%)	
HCV eradicated			
Yes	254 (98.1%)	99.1(%)	0.7
No	5 (1.9%)	1 (0.9%)	
HIV-related parameters			
HIV co-infection			
Yes	148 (56.1%)	66 (60.0%)	0.5
No	116 (43.9%)	44 (40.0%)	
Undetectable HIV viral load*			
Yes	90 (85.7%)	48 (69.8%)	0.02
No	39 (14.3%)	8 (30.2%)	
HIV viral load (if detectable)* (log copies/mL)	2.587 (2.107–3.068)	3.038 (1.621–4.456)	0.4
CD4 lymphocytes* (cells/μL)	592.0 (533.3–650.8)	669.2 (585.1–753.2)	0.15
Laboratory blood parameters at baseline			
Hemoglobin (g/dL)	15.04 (14.83–15.24))	13.98 (13.74–14.23)	< 0.0001
Leukocytes (cells/μL)	6766 (6453–7079)	6251 (5877–6626)	0.06
Platelets (× 1000/μL)	172.2 (164.0–180.3)	192.5 (177.8–207.2)	0.01
Aspartate aminotransferase (U/L)	70.0 (63.4–76.6)	67.2 (51.8–82.5)	0.7
Alanine aminotransferase (U/L)	81.3 (72.2–90.4)	66.3 (53.6–79.0)	0.07
Alkaline phosphatase (U/L)	90.8 (86.2–95.5)	90.5 (84.5–96.5)	0.9
γ-Glutamyl transferase (U/L)	170.1 (124.4–215.9)	88.5 (60.4–116.6)	0.03
Total bilirubin (mg/dL)	0.996 (0.931–1.061)	0.873 (0.802–0.943)	0.03
Total proteins (g/dL)	7.638 (7.509–7.667)	7.572 (7.437–7.707)	0.5
Albumin (g/dL)	4.301 (4.240–4.362)	4.231 (4.146–4.317)	0.2
Fibrinogen (mg/dL)	367.6 (350.5–384.7)	365.4 (340.3–390.6)	0.9
INR	1.077 (1.045–1.110)	1.038 (1.008–1.069)	0.15
Glucose (mg/dL)	106.8 (101.4–112.3)	100.1 (92.2–108.0)	0.17
Urea (mg/dL)	35.5 (33.9–37.1)	33.2 (31.1–35.2)	0.09
Creatinine (mg/dL)	0.882 (0.858–0.906)	0.772 (0.736–0.808)	< 0.0001
Total cholesterol (mg/dL)	164.7 (159.8–169.6)	180.8 (172.4–189.2)	0.0007
HDL cholesterol (mg/dL)	46.0 (43.7–48.3)	57.8 (53.7–61.9)	< 0.0001
LDL cholesterol (mg/dL)	93.8 (88.9–98.8)	98.2 (89.9–106.5)	0.4
Fibrosis parameters at baseline			
Transient elastometry (kPa)	11.21 (10.26–12.24)	8.77 (7.71–9.97)	0.003
APRI index	1.04 (0.93–1.17)	0.79 (0.65–0.96)	0.01
Forns index	6.47 (6.19–6.75)	5.44 (5.03–5.88)	< 0.0001
FIB-4 index	2.21 (2.01–2.43)	1.90 (1.64–2.20)	0.08
Stage of liver fibrosis (measured by TE)			
F0–F1	78 (29.7%)	47 (42.7%)	0.02
F2	50 (19.0%)	26 (23.6%)	
F3	48 (18.3%)	13 (11.8%)	
F4	87 (33.1%)	24 (21.8%)	
Change in fibrosis parameters after 24 months			
Absolute change in TE^‡^ (kPa)	− 6.09 (− 8.02, − 4.16)	− 3.05 (− 4.45, − 1.64)	0.07
Absolute change in APRI*^‡^	− 1.15 (− 1.45, − 0.85)	− 1.08 (− 1.71, − 0.45)	0.8
Absolute change in Forns^‡^	− 1.37 (− 1.58, − 1.16)	− 1.19 (− 1.52 to 0.86)	0.4
Absolute change in FIB-4^‡^	− 1.16 (− 1.48, − 0.83)	− 0.67 (− 1.36, − 0.02)	0.16
Relative improvement in TE^§^ (%)	36.0% (40.9–30.7)	27.5% (35.3–18.8)	0.1
Relative improvement in APRI^§^ (%)	61.4% (66.5–55.6)	59.1% (69.0–45.9)	0.7
Relative improvement in Forns^§^ (%)	21.2% (24.5–17.8)	21.2% (26.6–15.5)	1
Relative improvement in FIB-4^§^ (%)	32.2% (37.4–26.6)	23.2% (35.5–8.5)	0.14

Values are expressed as mean (95% CI) or % as appropriate.

DAA denotes direct acting antivirals, HCV hepatitis C virus, HIV human immunodeficiency virus, HDL high density lipoproteins, LDL low density lipoproteins, INR international normalized ratio, TE transient elastometry.

*Only in HIV-infected patients.

^‡^Difference and ^§^ratio between 24-month and baseline intra-subject measurements.

Regarding LF issues, men had significantly more advanced liver disease at baseline than women, as evaluated by the non-invasive markers, but there were not significant differences in the absolute or relative changes of these markers when the 24th-month was compared with the baseline values.

**Figure 1:**
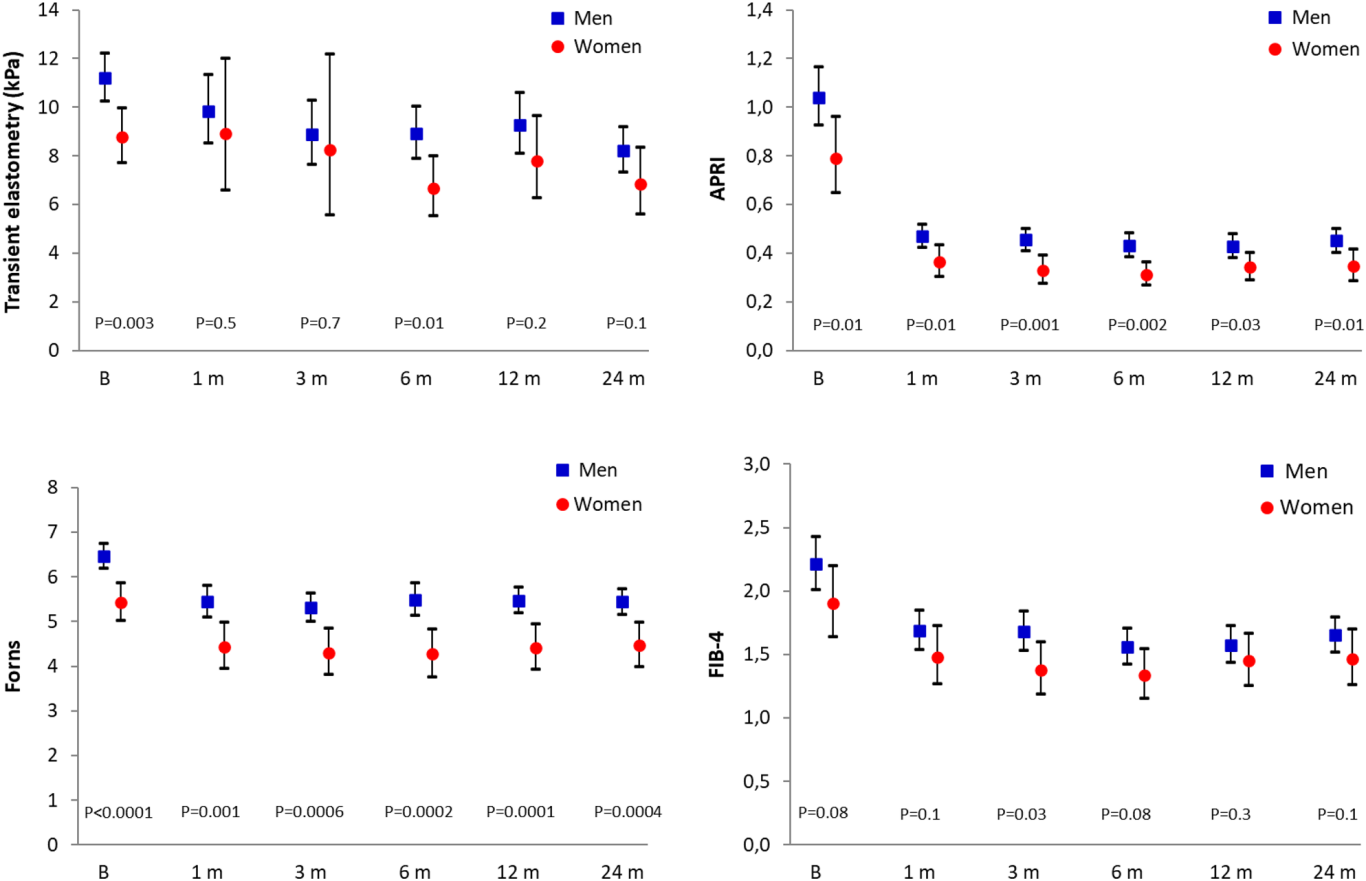
Course over time of the four non-invasive fibrosis indexes in men and women (mean, 95% CI).

Figure 1 shows the course over time of the four non-invasive indexes studied. The baseline differences between men and women were maintained during follow-up, depicting therefore parallel curves. The figure also shows a downward course over time, which resulted in statistically significant differences between the intra-subject final and initial measurements for each marker in both sexes (P < 0.05 for each comparison). Therefore, men and women had a favorable and similar short and long term fibrosis responses to DAA therapy in univariate analysis.

**Figure 2:**
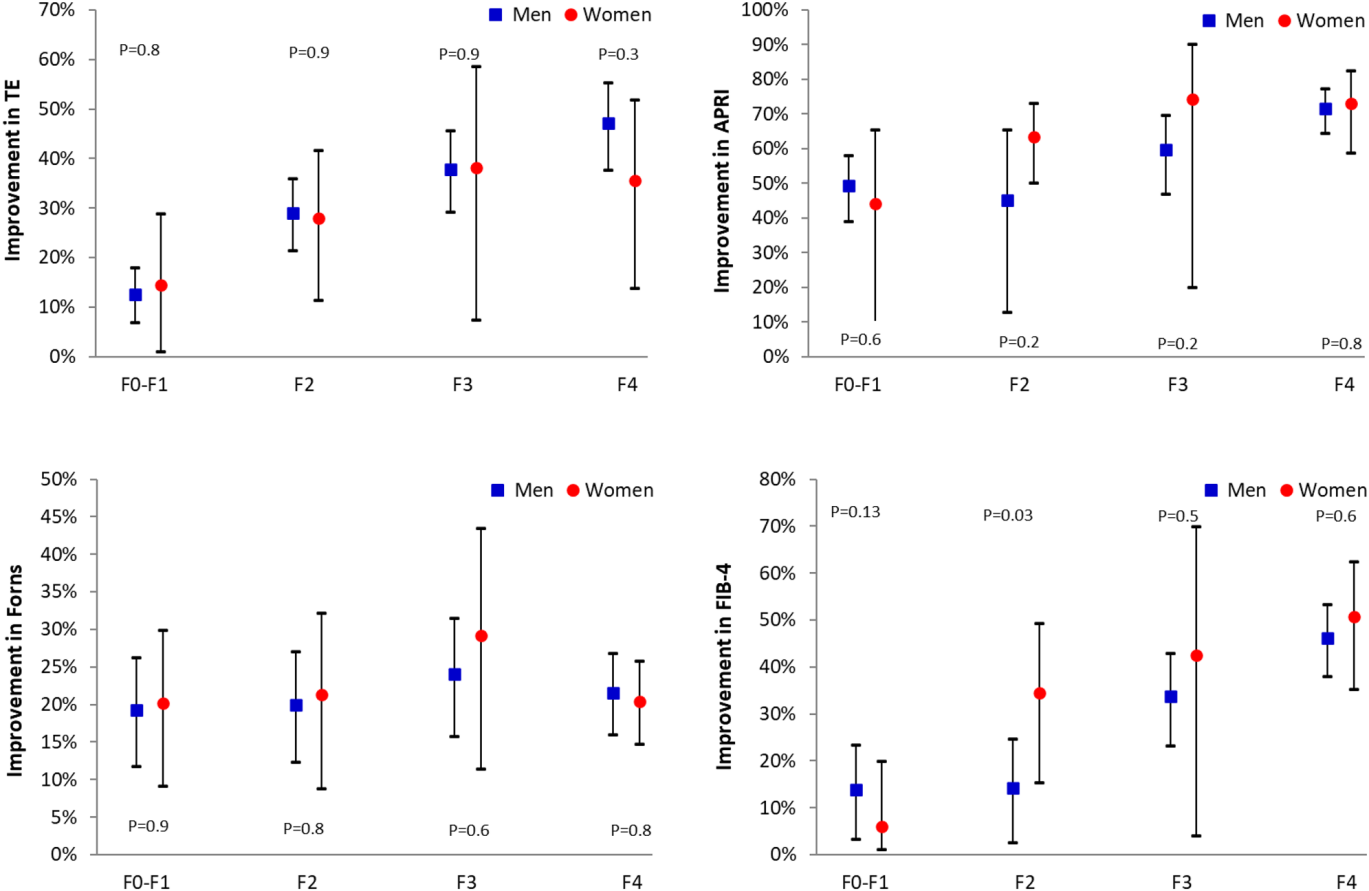
Relative improvement of markers of fibrosis at 24 months as compared with the baseline measurement according to the baseline fibrosis stage (mean, 95% CI).

Figure 2 shows the relative improvement in fibrosis markers at 24 months vs the initial measurements according to the baseline fibrosis stage. It can be appreciated that the degree of improvement was directly related to the baseline degree of fibrosis, experiencing higher reductions in fibrosis those patients with more advanced liver disease. Likewise, there were no significant differences between sexes, indicating similar fibrosis improvements in men and women regardless of the fibrosis stage.

**Figure 3:**
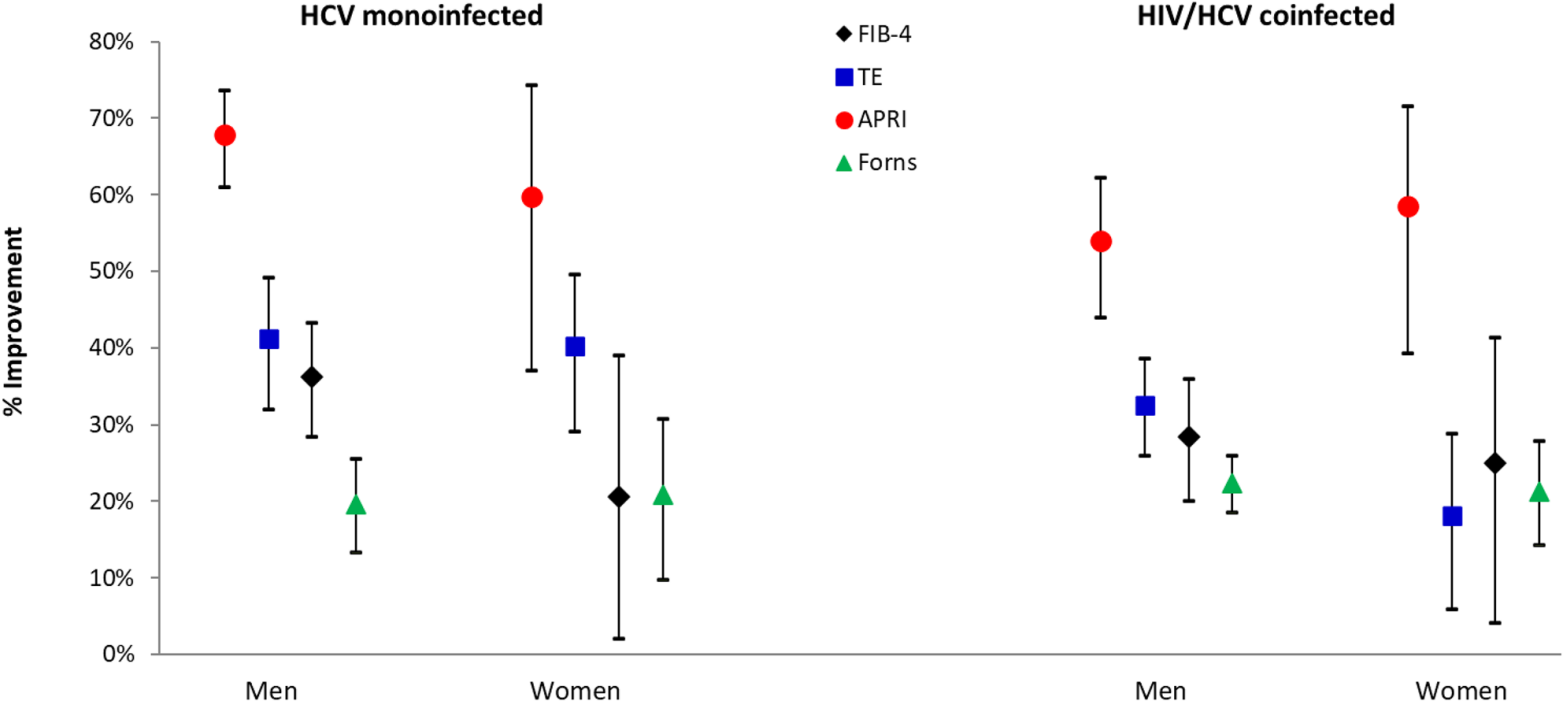
Relative improvement of markers of fibrosis at 24 months as compared with the baseline measurement according to the HIV/HCV status (mean, 95% CI).

Figure 3 depicts the relative improvements respect to the baseline values of the four markers in men and women according to the presence or absence of HIV co-infection. The fibrosis changes after DAA therapy were similar in monoinfected and in coinfected patients, and were also similar in both sexes for all markers (P = 0.08 to P = 0.9), with the only exception of TE in HCV/HIV coinfected patients, as women had somewhat lower improvements than men (difference between the means 14.4%, P = 0.03).

Finally, multivariate general linear models were constructed to evaluate the course over time of the four non-invasive markers of fibrosis, including variables that potentially could influence the degree and evolution of fibrosis, such as age, HIV infection, type, duration and virological results of DAA therapy, HCV viral load, and baseline fibrosis stage. Similarly to the univariate analysis described in Fig. 1, the downward course of the curves of men and women generated by the multivariate model were markedly parallel for all markers, indicating that gender did not influence the course of fibrosis as evaluated by these four markers: TE (P = 0.8), APRI (P = 0.9), Forns (P = 0.4), and FIB-4 (P = 0.7).

## Discussion

Our study showed a continuous decline in the long term in LF assessed by 4 NILFM (TE, APRI, Forns, FIB-4) in HCV infected patients after DAA therapy with parallel LF curves in men and women and no gender differences. Men had significantly more advanced LF at baseline compared to women. However fibrosis improvement curves went in parallel in both sexes throughout the 24 month follow up with independence of the baseline fibrosis stage. No differences among sexes in the comparison of LF decline course after DAA treatment between HCV/HIV coinfected individuals were detected either. Our results suggest that gender does not play any significant role in the evolution of LF after DAA therapy in HCV-mono and HCV/HIV coinfected individuals.

We confirmed previous reports that HCV-monoinfected and HCV/HIV-coinfected male had higher LF at baseline compared to women^14,18^. Previous works have suggested a protective effect of estrogens on fibrogenesis by the inhibition of stellate cells^33,34^. Furthermore, low plasma estrone levels associated with increased disease severity in women with HCV-related liver cirrhosis^35^. The mean age of the women included in our study, 51.63 years, implied that they were already postmenopausal or near menopause and therefore with low estrogens plasma levels. However, any possible relationship between LF and hormone levels should be cautious, taking into account the substantial differences in sexual hormone levels between men and women and, even within each gender, the marked hormonal changes related to age, menopause, phase of menstrual cycle and severity of liver fibrosis. Females are more vulnerable to ethanol compared to male because their smaller volume of distribution and reduced gastric alcohol dehydrogenase activity^36^. However the increased LF we observed in men was independent of ethanol abuse, usually more prevalent in male, because patients with ethanol consumption ≥ 50 g/day for > 5 years were excluded from the study. Although it could be arguable the use of the same cut-off in men and women, no specific cut-off has been reliably defined for women and, in addition, alcohol did not seem to play any relevant deleterious role in women according to our observations, as the fibrosis course was similar and parallel from different perspectives, and men had baseline worse fibrosis values than women.

This men-associated LF enhancing effect was also independent of the presence of HIV-coinfection, and of its course assessed by CD4 levels and HIV viral load. Although we recorded data about HIV therapy, the multiplicity of drugs and regimens, both at baseline and during follow-up, as well as the fact that treatment regimens were composed of more than one drug, preclude the analysis of HIV-related drugs. In addition, it was independent of the HCV features assessed by HCV viral load and genotype.

Very few studies have focused in the LF improvement assessment in HCV patients after DAA treatment with an extended follow-up. Even more rarely these studies have focused on HCV-infected women mostly because drug abuse, the most current HCV transmission mechanism in developed countries, is less prevalent in women. However, all the HCV-infected women and men included in our study were former IV drug users. Gardner et al. report an early steep decline in LF measured by biochemical fibrosis markers (ELF, APRI, FIB-4) followed by a surprising flattening of the LF decline in 116 HCV/HIV-infected American women 1–2 years after HCV therapy^26^. This long-term flattening curve observation was more evident for ELF score than for the other fibrosis biomarkers. ELF score is a marker of extracellular modeling used instead of TE in Gardner´s work. ELF score is derived from hyaluronic acid, amino-terminal propeptide of type III collagen (PIIINP) and tissue inhibitor of matrix metalloproteinase 1 (TIMP-1). The authors argued that were was little LF regression at long-term after HCV-therapy or that ELF score gave not an ideal reflection of fibrosis but rather liver inflammation^26^. We observed a continuous progressive LF curve decline in our study. However we assessed LF with TE, APRI, Forns and FIB-4 but not with the ELF score. Anyhow in a previous paper derived from the same study we observed a progressive decline in TIMP-1 plasma levels, a biochemical marker included in the ELF score after 2 year follow-up in HCV and HCV/HIC coinfected patients treated with DAA^25^. On the other hand, the findings of Gardner et al.^26^ about the improvement in APRI and FIB-4 markers within one year post-therapy, but not in the second year, may be explained and complemented by our findings based on shorter interval determinations. Thus, we observed a marked improvement in APRI and FIB-4 indexes during the first month of therapy, reaching a plateau afterwards. As AST and ALT are used for calculations of these indexes, an early and marked improvement in inflammation would be responsible for these observations. In fact, we analyzed the course of AST and ALT over time and, as expected, we found a marked decrease during the first month of therapy, averaging normal levels at this time point, followed by a flattening during the remaining follow-up (data not shown). These findings support the role of rapidly decreasing inflammation after the onset of therapy as the explanation of the early improvement, and further flattening, in these markers.

The strongest points of our study include its prospective nature, the very long follow-up after DAA therapy, the high number of patients enrolled, including 110 women, and the comprehensive evaluation at each time point. However there are also some limitations, including the lack of liver biopsies, the gold standard for fibrosis assessment. However, biopsies have several limitations including sampling error, cost, and risk of complications. Therefore liver biopsies are being replaced by TE, a technique we used along with other noninvasive biochemical markers of LF. Also, sexual hormone determinations were beyond the purpose of the study and were not available, but their interpretation would be difficult taking into account the large differences between men and women, and the great variability in their serum levels commented above, which make very difficult the drawing of reliable conclusions.

We did not assess sex hormone plasma levels in order to rule out a protective effect of estrogens on LF as other authors did^35^. Finally, the multiplicity and efficacy of DAA combinations preclude the evaluation of the effect of specific regimens, although this shortcoming does not affect the evaluation of the fibrosis response to DAA therapy.

We conclude that in a large HCV mono and HCV/HIV coinfected cohort in which ethanol abuse was excluded, LF regression after DAA, as evaluated by NILFM, seems to be independent of gender, even from the perspective of the stage of fibrosis at baseline, the HCV features, and the HIV coinfection.

## Data Availability

The datasets generated during and/or analyzed during the current study are not publicly available due to individual privacy but are available from the corresponding author on reasonable request.
